# Supplementary figures and images for: Effects of the pan-caspase inhibitor Q-VD-OPh on human neutrophil lifespan and function
Source: PLoS One. 2025 Jan 7;20(1):e0316912. doi: 10.1371/journal.pone.0316912 (PMC11706505; doi:10.1371/journal.pone.0316912)

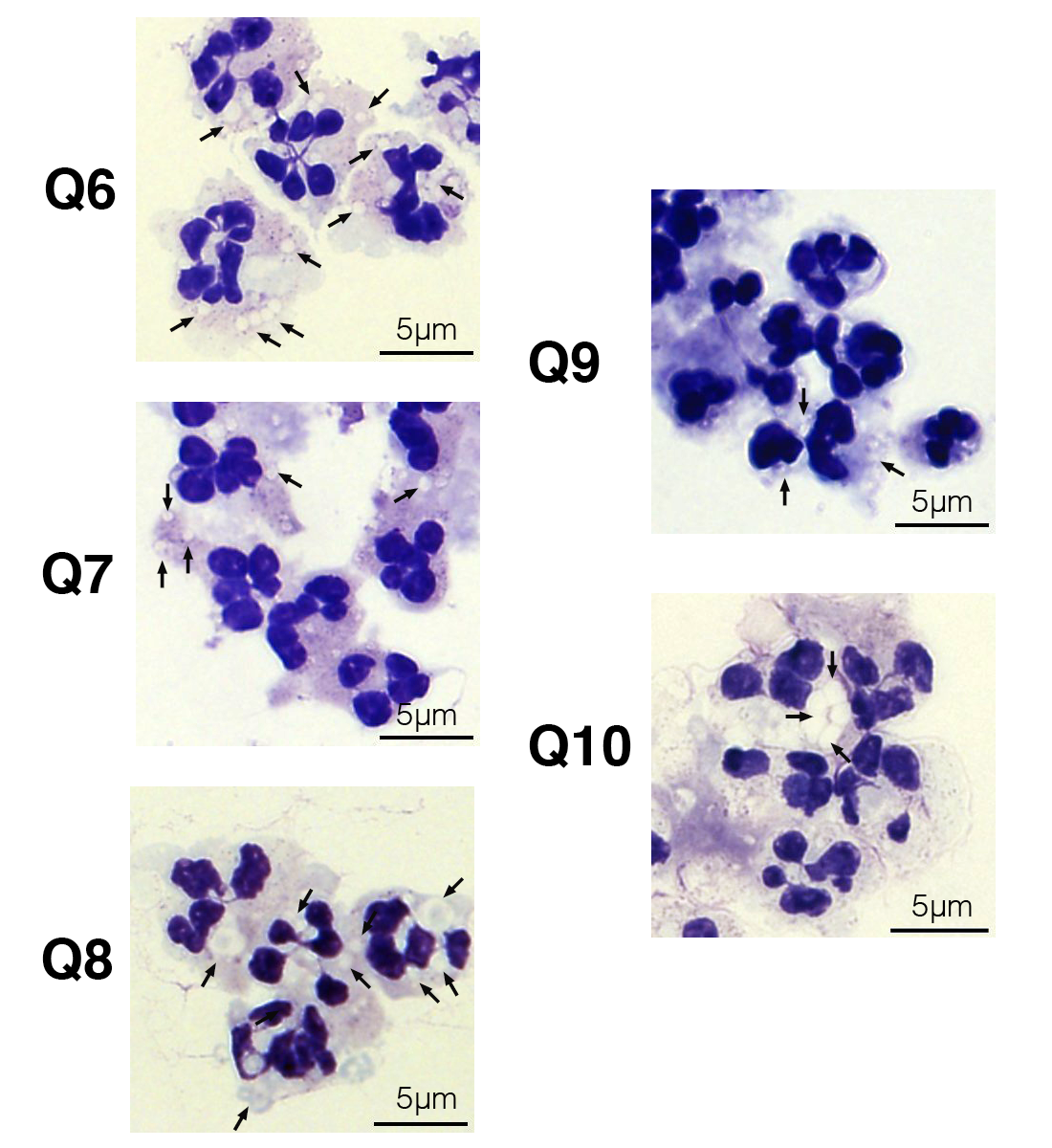

Supplement: S1 Fig — Representative Hema-3 images of human neutrophils that were incubated in medium containing 10 μM QVD for 6–10 days (Q6-Q10) and then stained with Hema-3 reagents. Arrows indicate cytoplasmic vacuoles. (TIF) [file pone.0316912.s001.tif]

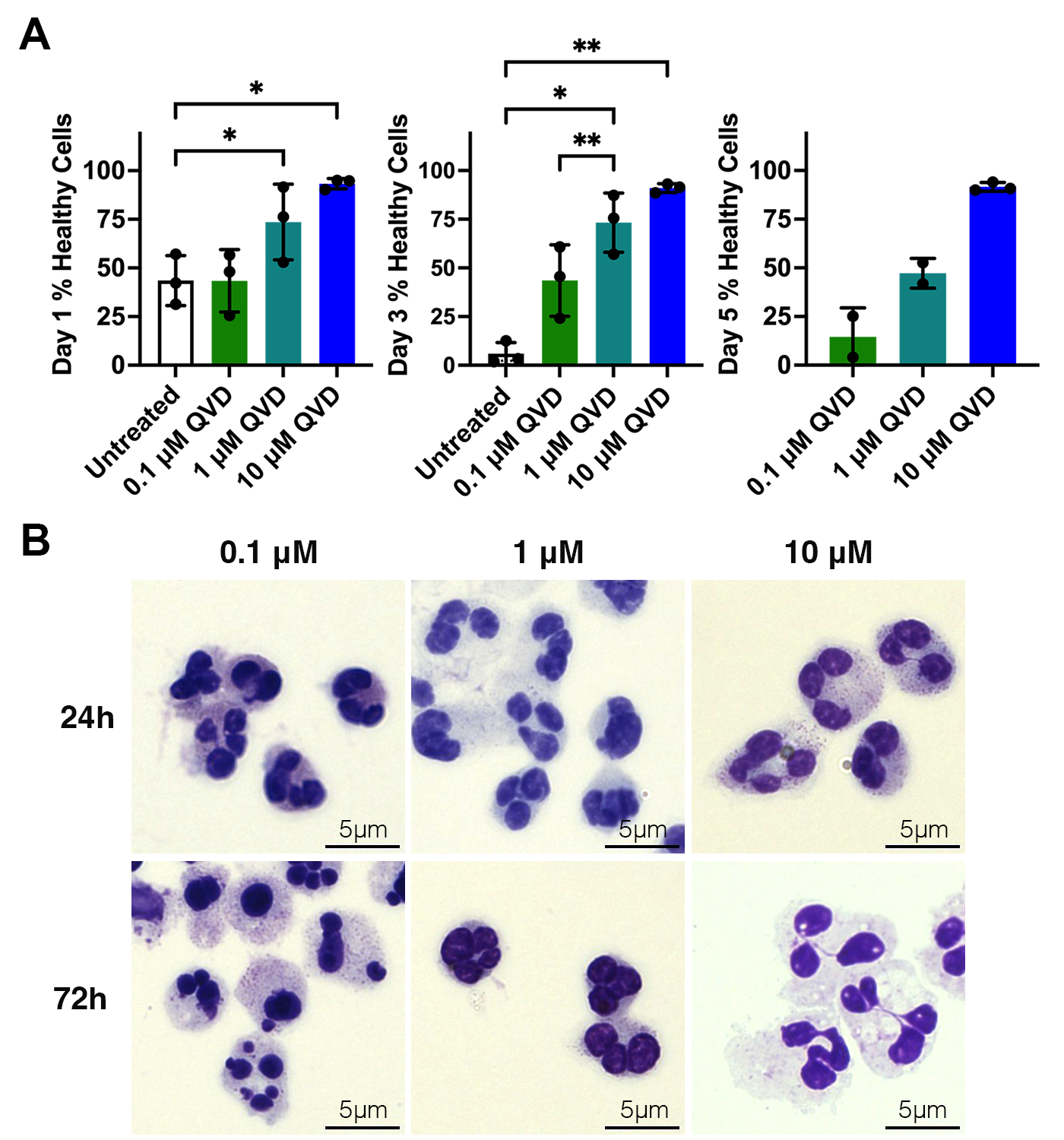

Supplement: S2 Fig — Freshly isolated neutrophils were aged in culture in the presence and absence of 0.1, 1 or 10 μM QVD, as indicated, prior to analysis. (A) Graphs show the percentage of healthy cells for each condition and time point as determined by Annexin V-FITC/PI staining and flow cytometry. Data are the average + SD from three independent experiments for days 1 and 3 and 2–3 experiments for day 5. Data from days 1 and 3 were analyzed by one-way ANOVA with Tukey’s multiple comparisons post-test. *p<0.05, **p<0.01. (B) Representative images of Hema-3-stained cells show the nuclear morphology of cells treated with the indicated concentrations of QVD for 24 or 72 h. (TIF) [file pone.0316912.s002.tif]

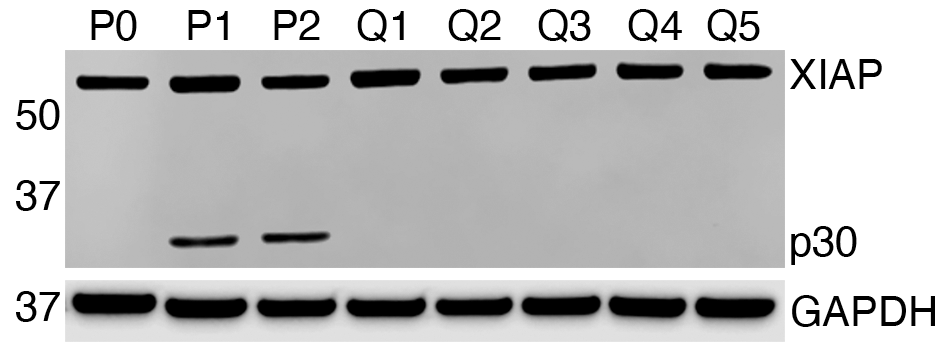

Supplement: S3 Fig — Immunoblot of neutrophil lysates prepared on days 0–2 (P0-P2) or after 1–5 days of QVD treatment (Q1-Q5) probed to detect XIAP with GAPDH as the loading control. A p30 XIAP fragment indicative of apoptosis was present in aged PMNs on days 1 and 2 but was absent in freshly isolated cells and cells treated with QVD for 1–5 days. (TIF) [file pone.0316912.s003.tif]

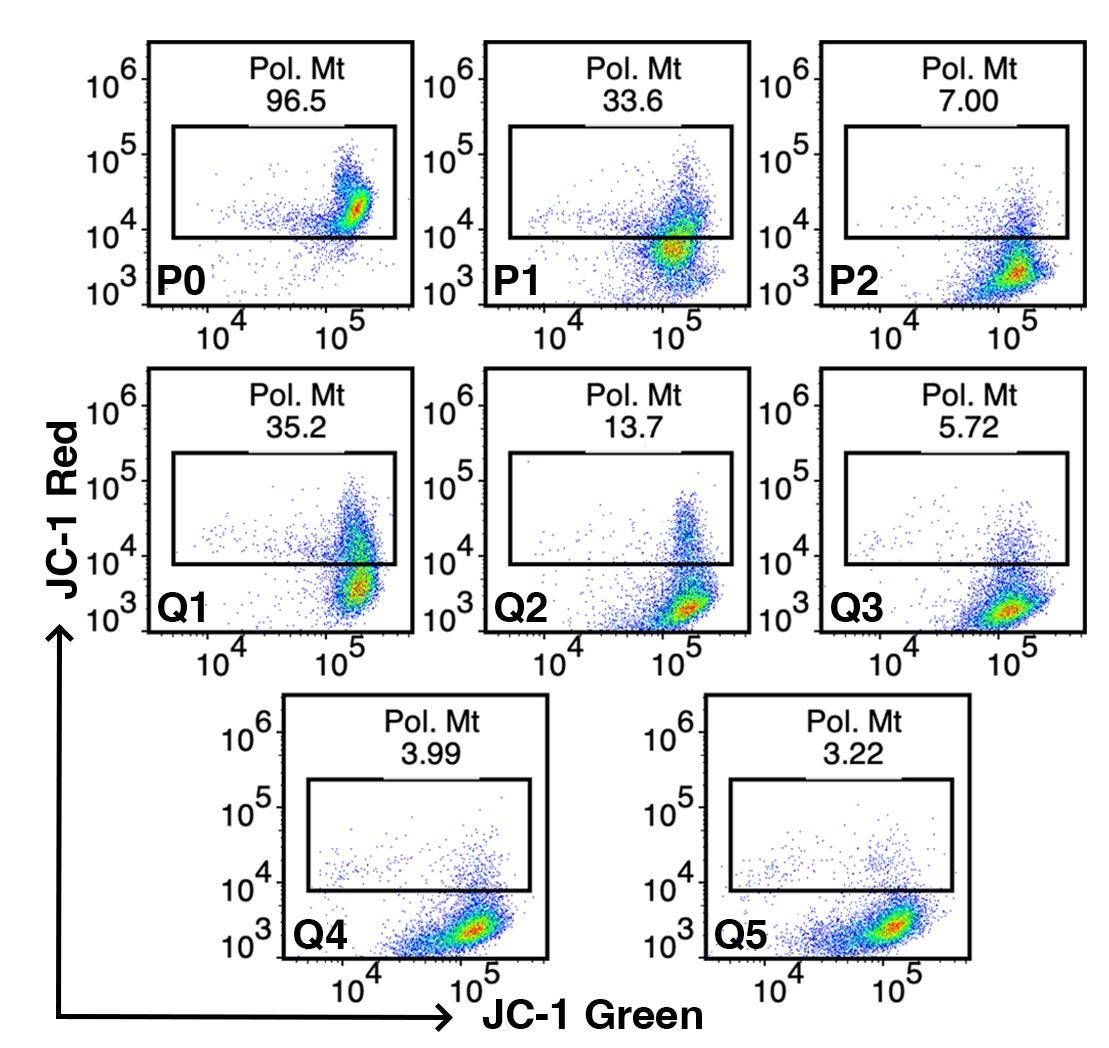

Supplement: S4 Fig — Neutrophils were stained with MitoProbe™ JC-1 and analyzed via flow cytometry at each time point. P0-P2, freshly isolated, 24 h and 48 h-aged neutrophils, respectively. Q1-Q5, neutrophils treated for 1–5 days with Q-VD-OPh. (TIF) [file pone.0316912.s004.tif]

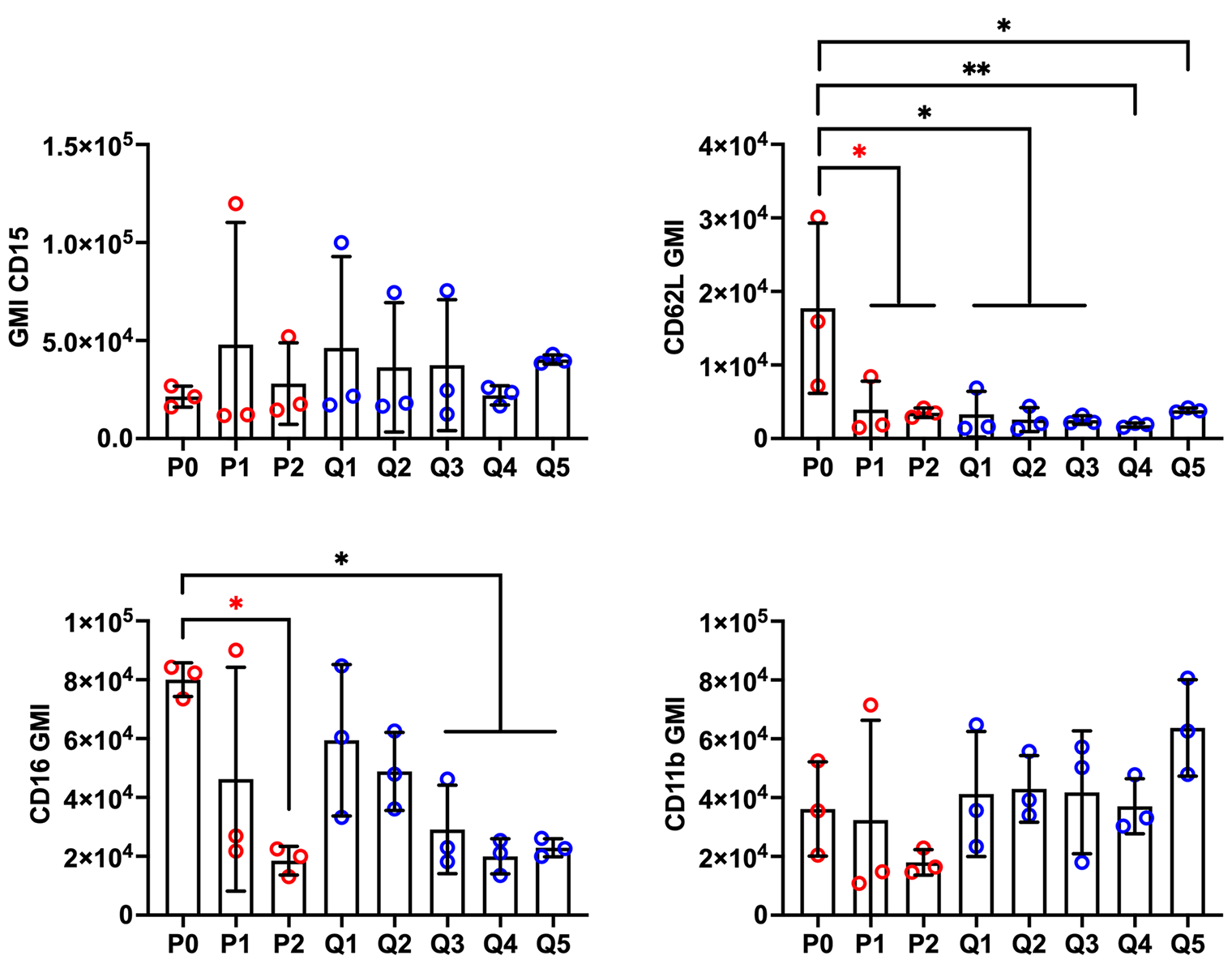

Supplement: S5 Fig — Freshly isolated neutrophils (P0), cells that were cultured in medium for 1–2 days (P1, P2) and cells that were incubated for up to 5 days in medium containing 10 μM QVD (Q1-Q5) were stained using the DURAClone IM Granulocytes kit and analyzed via flow cytometry. Pooled data from three independent experiments show the average geometric mean intensity (GMI) + SD for CD15, CD62L, CD16 and CD11b, as indicated. For all graphs data were analyzed by two-way ANOVA with Tukey’s multiple comparisons post-test. *p<0.05 and **p<0.01. (TIF) [file pone.0316912.s005.tif]

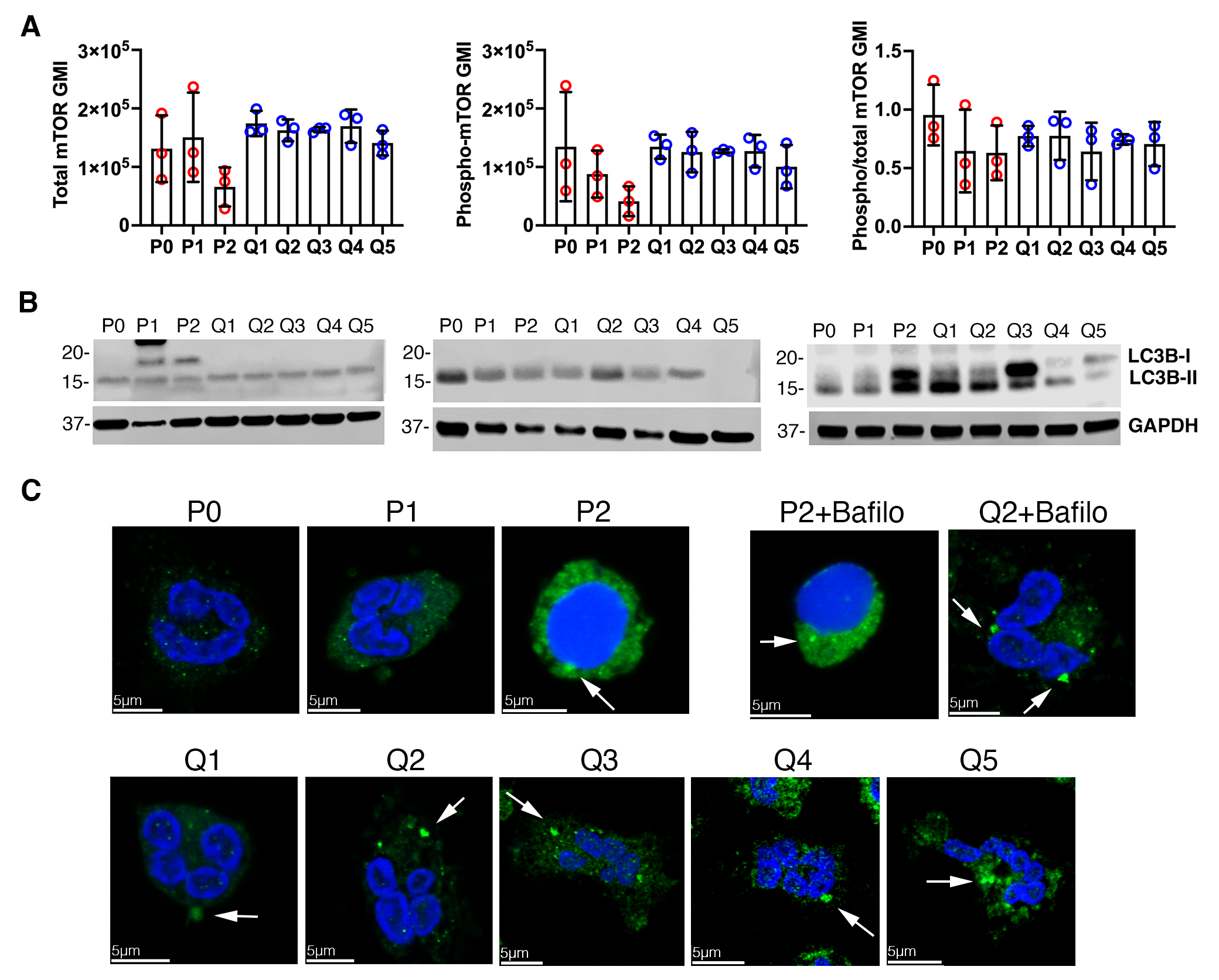

Supplement: S6 Fig — Freshly isolated (P0), 24–48 h aged cells (P1-P2) and cells treated for 1–5 days with Q-VD-OPh (Q1-Q5) were compared. (A) Graphs show the total mTOR, S2446-phosphorylated mTOR and the ratio of total/phosphorylated mTOR for each condition. Data are the mean ± SD of three independent experiments. (B) Western blots of cell lysates were probed to detect LC3B-I and LC3B-II with GAPDH as the loading control. Data for three independent experiments are shown. (C) Representative confocal images show p62 in green and nuclear DNA (DAPI) in blue. Arrows indicate p62-positive autophagosomes. (TIF) [file pone.0316912.s006.tif]
