# Supplementary material for: Effects of the pan-caspase inhibitor Q-VD-OPh on human neutrophil lifespan and function
Source: PLoS One. 2025 Jan 7;20(1):e0316912. doi: 10.1371/journal.pone.0316912 (PMC11706505; doi:10.1371/journal.pone.0316912)

Procaspase-3

Procas3 expected 34 kDa
Cas3 expected 12, 17 kDa
GAPDH expected 37 kDa

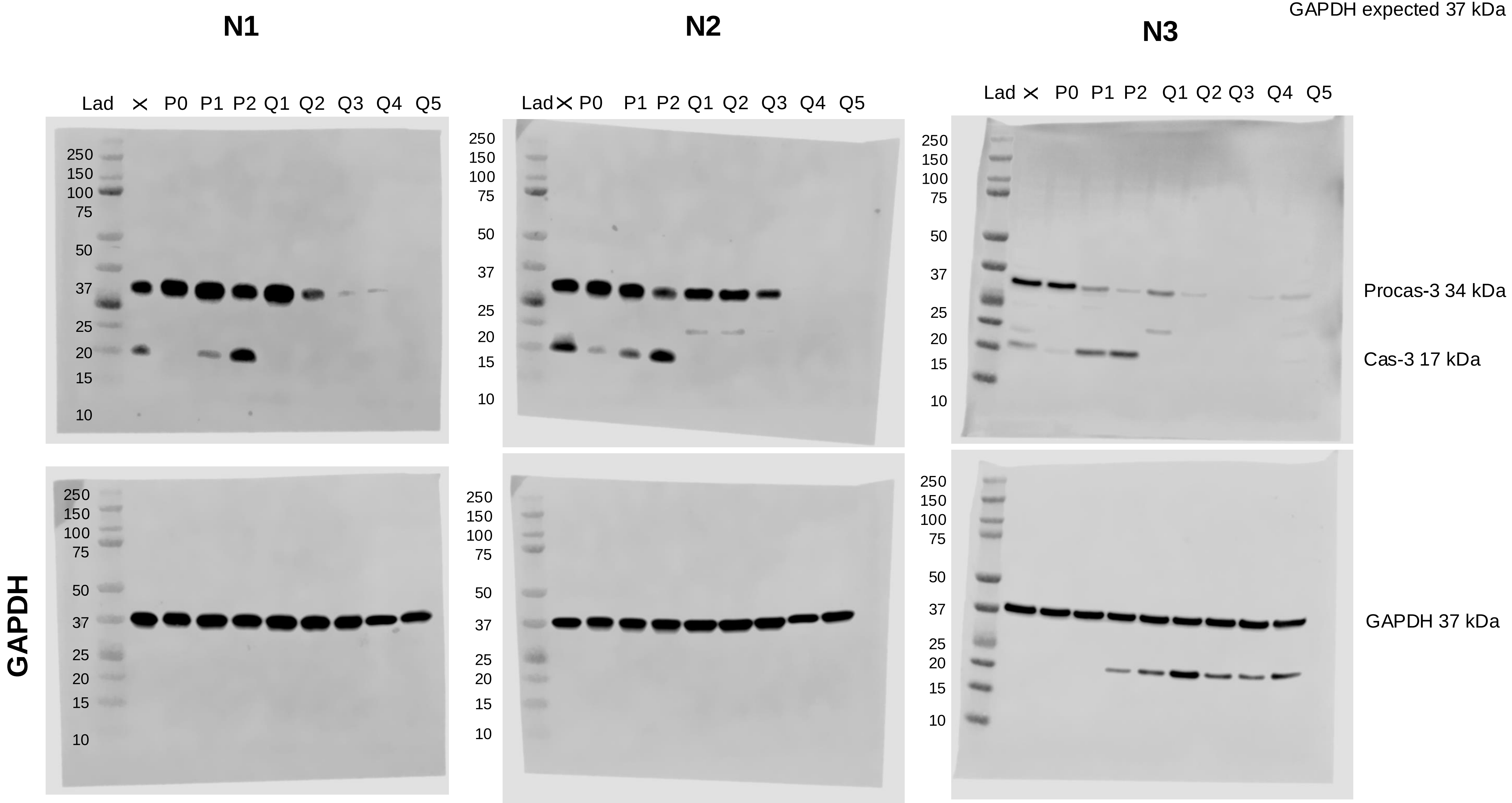

Procaspase-3 inhibitor treatment

Procas3 expected 34 kDa
Cas3 expected 12, 17 kDa
GAPDH expected 37 kDa

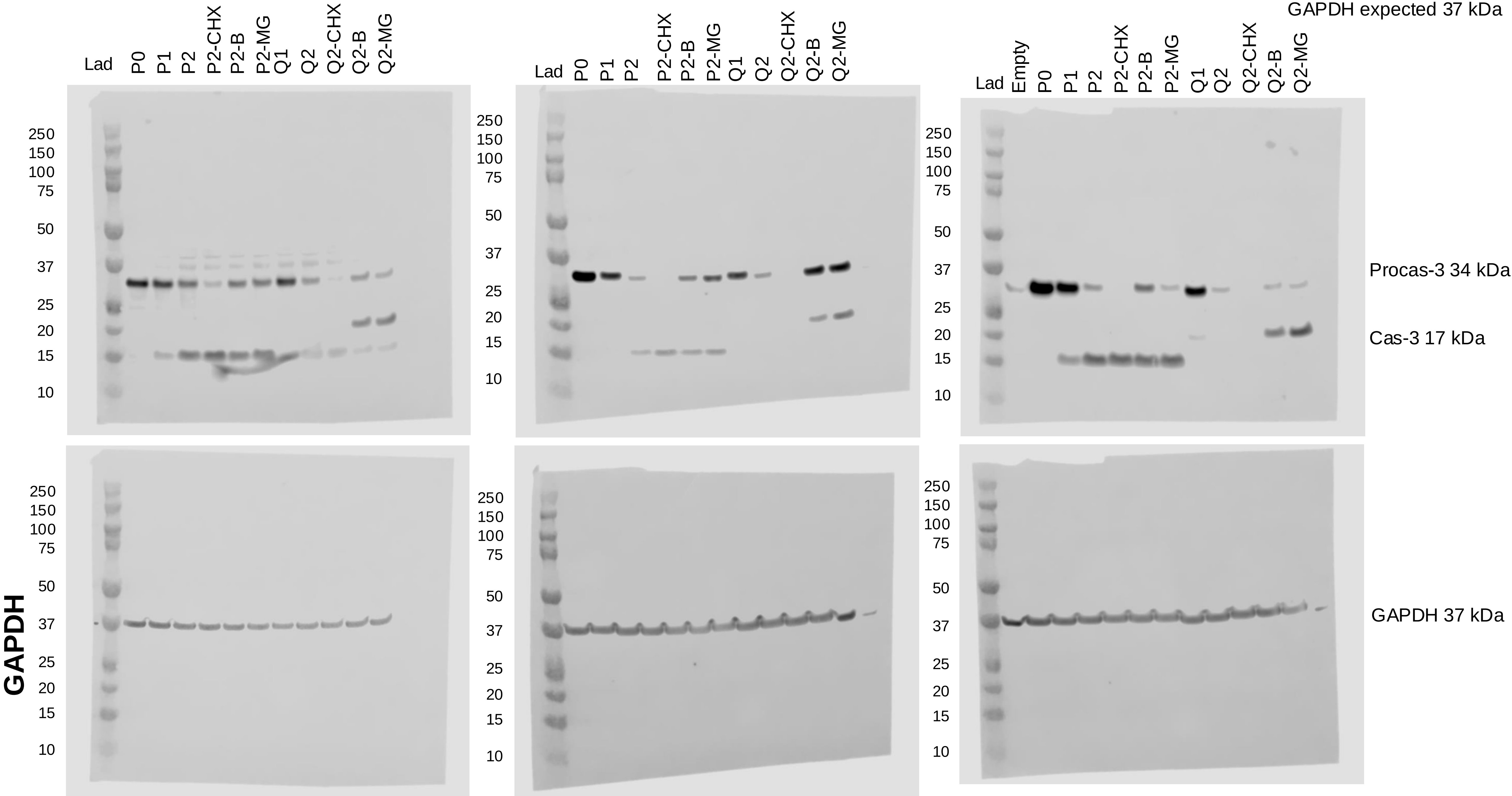

MCL1

MCL1 expected 37 kDa
GAPDH expected 37 kDa

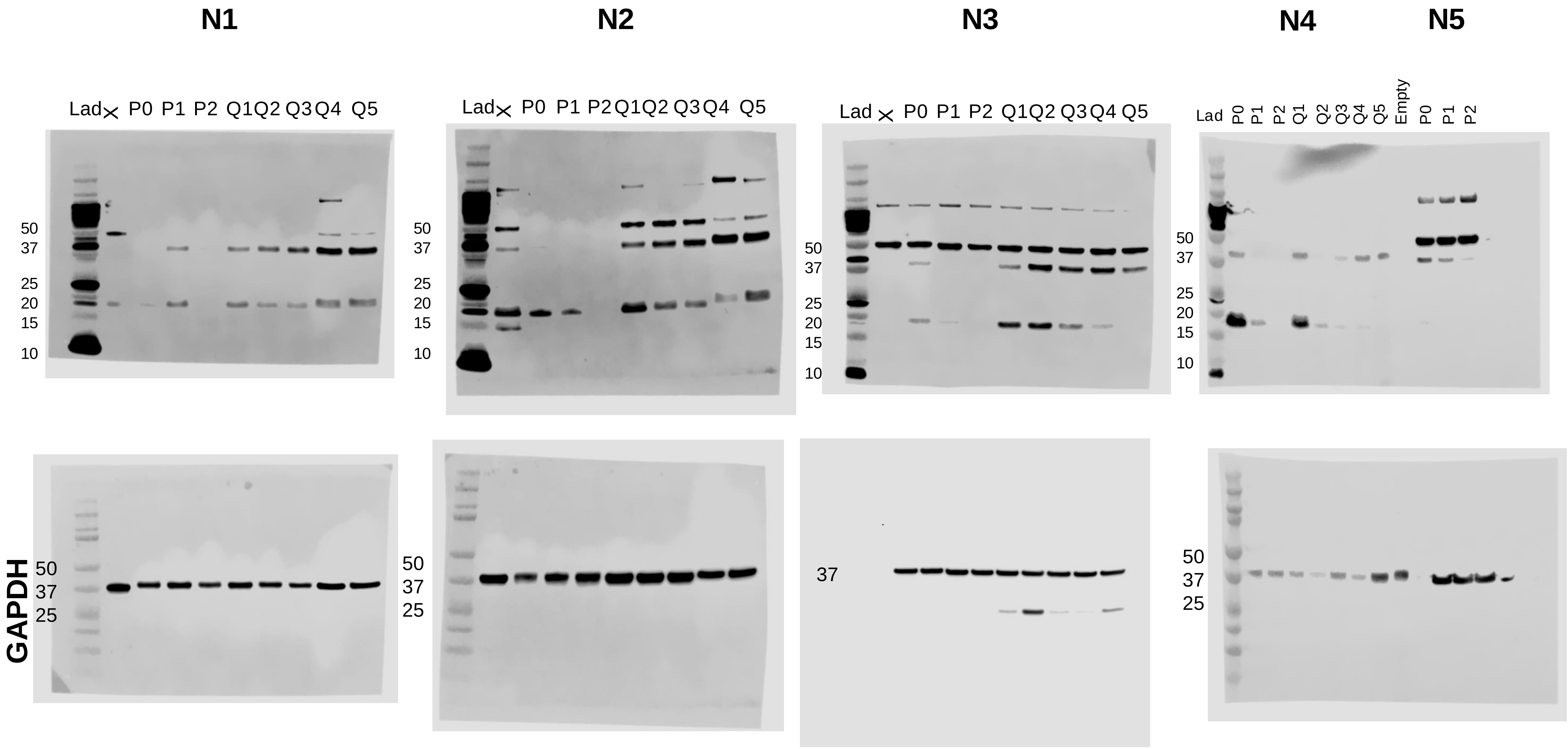

XIAP

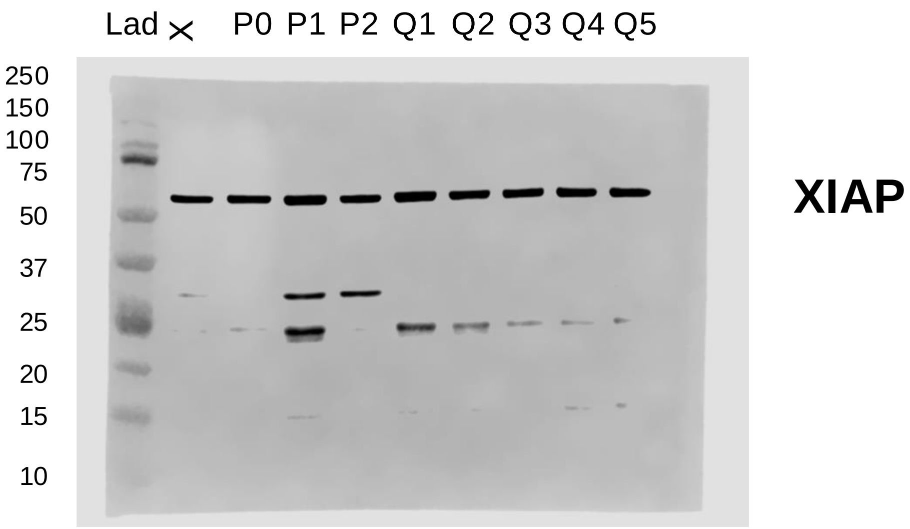

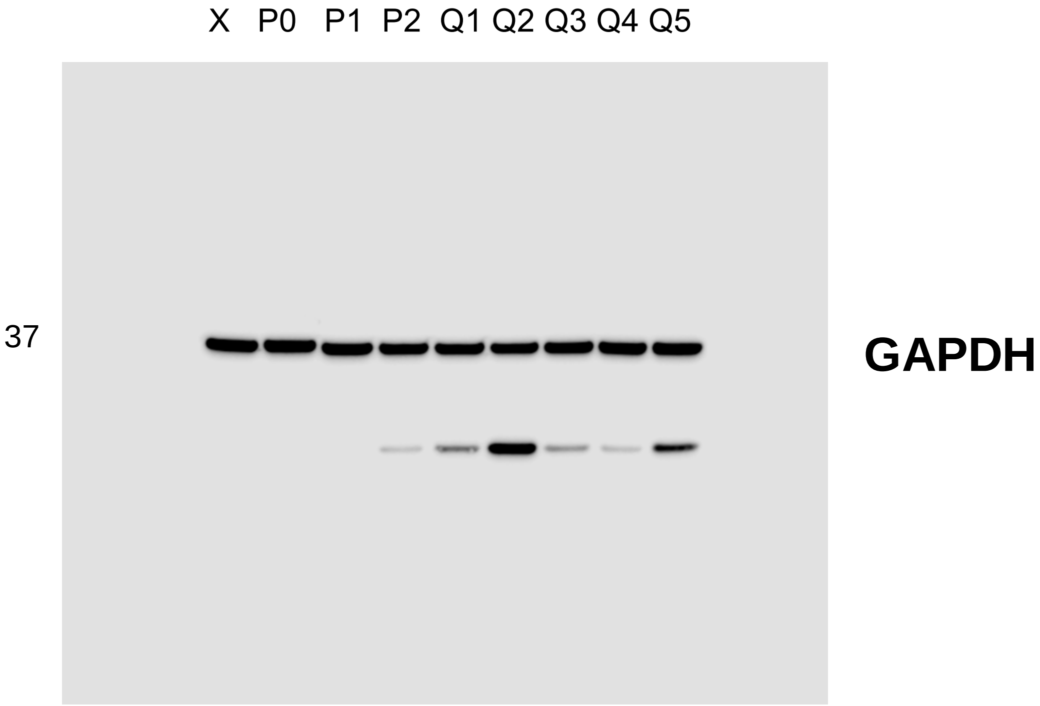

XIAP expected 53 kDa
GAPDH expected 37 kDa

LC3B

LC3BI expected ~16-18 kDa
LC3BII expected ~14-16 kDa
GAPDH expected 37 kDa

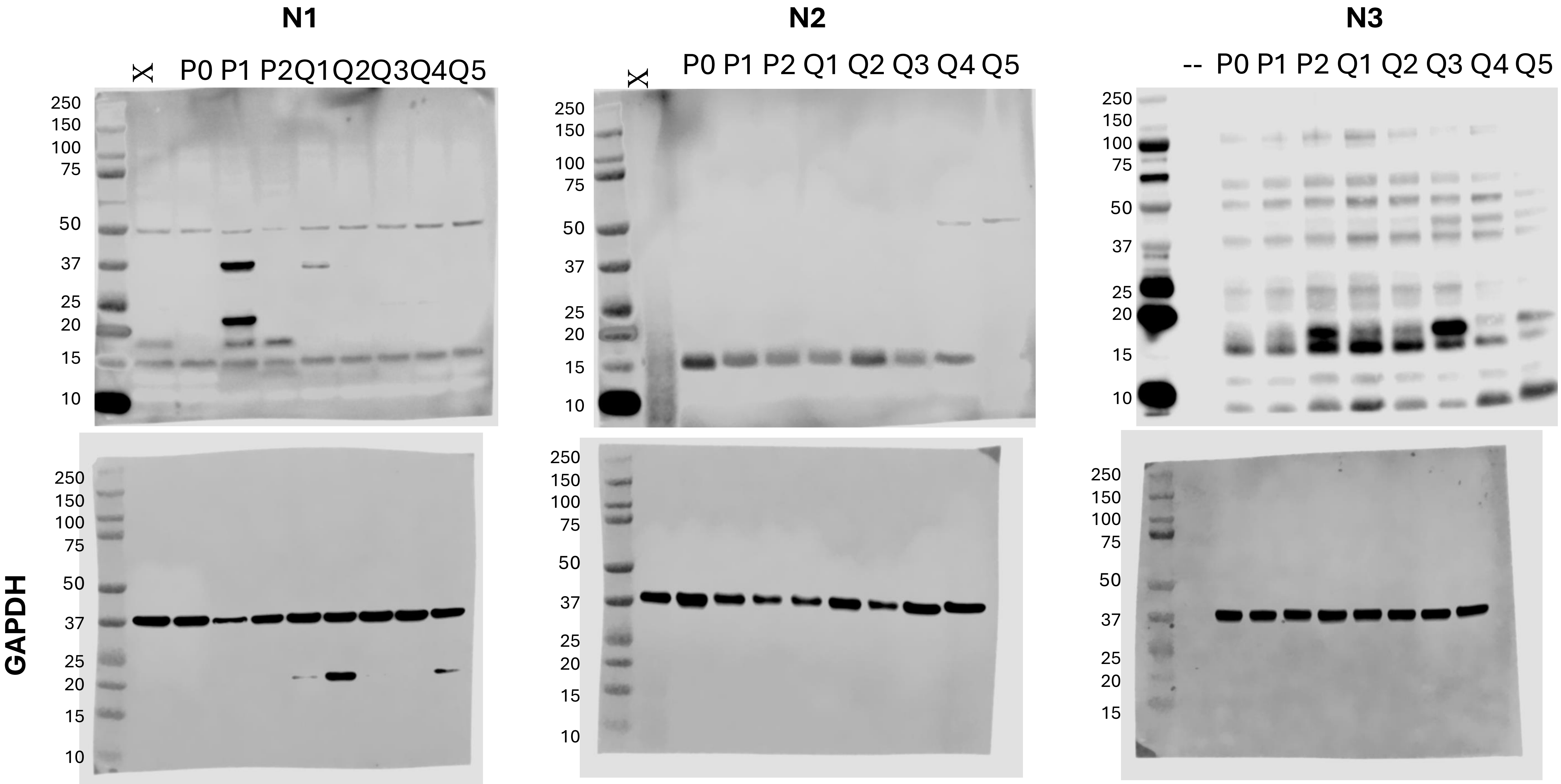

Supplement: S1 Raw images — (PDF) [file pone.0316912.s007.pdf]
